# Circulating fatty acid profiles and risk of pulmonary arterial hypertension: Evidence from a large prospective cohort study

**DOI:** 10.1097/MD.0000000000047645

**Published:** 2026-02-28

**Authors:** Shuo Liu, Haobei Yuan, Jingxian Cao, Liang Cai, Quanlei Wang, Xiaoping Zhou

**Affiliations:** a School of Traditional Chinese Medicine, Ningxia Medical University, Yinchuan, Ningxia, China.

**Keywords:** fatty acids, prospective study, pulmonary arterial hypertension, risk factors, UK Biobank

## Abstract

Fatty acids (FAs) have cardioprotective properties. However, their role in the development of pulmonary arterial hypertension (PAH) has not been thoroughly examined. This study aimed to investigate the relationship between circulating FA profiles and the risk of PAH. Data from the UK Biobank were used to assess the association between 8 circulating FAs and PAH risk. Cox proportional hazards regression models estimated hazard ratios and 95% confidence intervals, with stratified analyses conducted across demographic and clinical subgroups. Restricted cubic spline models were employed to explore potential nonlinear dose–response relationships, while accelerated failure time models assessed the effect of FA levels on the timing of PAH onset. Higher levels of all 8 FAs were linked to a reduced risk of PAH, with the strongest inverse associations observed for docosahexaenoic acid, omega-3, and polyunsaturated fatty acids (hazard ratio range: 0.76 to 0.85, all *P* < .001). Restricted cubic spline analysis revealed nonlinear relationships between docosahexaenoic acid, omega-3 FAs, and PAH risk. Additionally, accelerated failure time models showed that participants in the highest quartile of FA concentrations experienced a delay in PAH onset by approximately 60 to 120 months compared to those in the lowest quartile. Elevated circulating FA levels were associated with a lower risk of incident PAH, with consistent patterns across most demographic and clinical subgroups. These findings provide novel population-based evidence supporting the potential of FAs as biomarkers for PAH prevention and therapeutic targets.

## 1. Introduction

Pulmonary arterial hypertension (PAH) is a fatal cardiopulmonary vascular syndrome characterized by progressive pulmonary vascular remodeling, sustained increases in pulmonary arterial pressure and pulmonary vascular resistance, and eventual right heart failure and premature death.^[1]^ While the overall incidence of PAH in the general population remains low, its prevalence has steadily increased, and combined with its high mortality rate, PAH has become a significant public health concern.^[2]^ The pathophysiology of PAH involves a complex interplay of genetic factors, endothelial dysfunction, vascular remodeling, inflammation, and metabolic dysregulation.^[3–5]^ Emerging evidence suggests that metabolic abnormalities, particularly in lipid and fatty acid (FA) metabolism, may contribute to the initiation and progression of PAH by influencing vascular cell proliferation, inflammation, oxidative stress, and mitochondrial function.^[6,7]^

FAs, including saturated FAs (SFAs), monounsaturated FAs (MUFAs), and polyunsaturated FAs (PUFAs), are bioactive molecules that play critical roles in energy metabolism, membrane structure maintenance, signal transduction, inflammation, and vascular regulation.^[8–10]^ Previous studies have indicated that dysregulation of the FA profile, such as elevated SFAs and altered omega-3/omega-6 PUFA ratios, is linked to adverse cardiovascular outcomes, including atherosclerosis, heart failure, and systemic hypertension.^[11–13]^ Furthermore, Lin et al^[14]^ demonstrated in human studies that FAs, as novel vasoactive substances, may modulate cerebral blood flow and provide neuroprotection following ischemia. However, the role of circulating FAs in PAH remains poorly understood. Most existing evidence stems from small-scale observational or experimental studies, which are often limited by sample size, single-center cohorts, or cross-sectional designs.

The advent of large-scale, deeply phenotyped population cohorts has created new opportunities to uncover the metabolic mechanisms underlying rare cardiovascular diseases (CVDs) like PAH. The UK Biobank (UKB), a prospective cohort with over 500,000 participants and extensive lifestyle, clinical, biochemical, and genetic data, provides a powerful platform for exploring potential associations between circulating metabolites and disease risk.

This study leveraged metabolomics data from the UKB, obtained through nuclear magnetic resonance (NMR) spectroscopy and mass spectrometry, to investigate the relationship between circulating FAs and their subgroups with the risk of PAH. This approach allows for a more detailed characterization of lipid metabolic signatures relevant to PAH onset and offers new insights into the metabolic pathways potentially involved in its pathogenesis. These findings could inform the development of targeted PAH prevention strategies and strengthen the evidence linking lipid metabolism to pulmonary vascular disease.

## 2. Materials and methods

### 2.1. Study population

The UKB is a large, prospective, population-based cohort study that enrolled over 500,000 participants aged 37 to 73 years from across the United Kingdom between 2006 and 2010, with ongoing follow-up. All participants provided written informed consent, and the study was approved by the Northwest Multicenter Research Ethics Committee.^[15]^

### 2.2. Assessment of exposure

FA measures were obtained from baseline plasma samples analyzed using the NMR-based metabolomics platform developed and operated by Nightingale Health Ltd. (Helsinki, Finland). This standardized NMR platform quantifies the absolute concentrations (mmol/L) of multiple FA subtypes from a single non-fasting EDTA plasma sample. Detailed procedures for sample handling and analysis are outlined in the UKB metabolomics technical documentation. The present study included 8 FA-related biomarkers: docosahexaenoic acid (DHA), linoleic acid (LA), MUFAs, omega-3 FAs, omega-6 FAs, PUFAs, SFAs, and total FAs.

### 2.3. Covariates

Baseline sociodemographic, lifestyle, and clinical characteristics were collected through touchscreen questionnaires, nurse-led interviews, and physical measurements and were used as covariates. These variables included age, sex, ethnicity, body mass index (BMI), smoking status, alcohol consumption, Townsend Deprivation Index (TDI), physical activity, dietary score, history of chronic diseases (e.g., hypertension, diabetes mellitus [DM], CVD, chronic pulmonary diseases), and regular medication use (e.g., lipid-lowering agents, antihypertensive drugs, and antidiabetic medications). Physical activity was quantified in metabolic equivalent (MET) minutes.^[16]^ The TDI was used as a measure of socioeconomic status.^[17]^ The dietary score, ranging from 0 to 9, was derived from frequency of dietary intake, with higher scores indicating poorer dietary habits.^[18]^ CVD and chronic pulmonary diseases were diagnosed based on International Classification of Diseases, Tenth Revision (ICD-10) codes from the UKB. Specifically, CVD included heart failure (I50, I11.0, I11.9, I13), valvular heart disease (I34–I39), and coronary heart disease (I20–I25). Chronic pulmonary diseases primarily included chronic obstructive pulmonary disease (J44), bronchiectasis (J47), and interstitial pulmonary diseases (J84).

### 2.4. Ascertainment of outcomes

PAH diagnoses were confirmed using ICD-10 codes I27.0 and I27.2. The follow-up period for each participant began at the baseline assessment date and ended at the earliest occurrence of any of the following: first diagnosis of PAH, death, or October 27, 2022.

### 2.5. Statistical Analysis

Table S1, Supplemental Digital Content, https://links.lww.com/MD/R394 presents the number and proportion of missing values for each baseline covariate, which were handled using multiple imputation by chained equations with a random forest algorithm. Continuous variables are expressed as mean ± standard deviation (SD), where appropriate, and compared between groups using the independent-samples *t*-test or the Mann–Whitney *U* test, based on normality. Categorical variables are summarized as counts and percentages (N, %) and compared using the χ^2^ test or Fisher exact test.

FA concentrations were standardized using z-scores before analysis. Cox proportional hazards regression models were employed to estimate hazard ratios (HRs) and 95% confidence intervals (CIs) for the association between each 1-SD increase in FA levels and the risk of incident PAH. The proportional hazards assumption was tested using Schoenfeld residuals, and no violations were detected. Two models were constructed: Model 1 was adjusted for age, sex, and race; Model 2 was further adjusted for TDI, BMI, smoking status, DM, hypertension, use of antihypertensive medications, lipid levels, use of antidiabetic medications, alcohol consumption, dietary score, MET, and history of CVD and chronic pulmonary diseases.

Additionally, FAs were categorized into quartiles, with the lowest quartile (Q1) serving as the reference group. Kaplan–Meier survival curves were generated to compare the cumulative incidence of PAH across FA quartiles, and the log-rank test was used to assess differences. To further examine potential dose–response relationships between FA levels and PAH risk, restricted cubic spline analyses were performed, with 3 knots placed at the 10th, 50th, and 90th percentiles. Non-linearity was tested using the likelihood ratio test.^[19]^ To investigate the association between FA levels and median time to PAH onset, accelerated failure time models assuming a Weibull distribution were applied, adjusted for the same covariates as Model 2. Negative coefficients indicated a delay in PAH onset, while positive coefficients indicated an earlier onset.^[20]^

Subgroup analyses were performed, stratified by sex, age, race, BMI, smoking status, alcohol consumption, hypertension, DM, CVD, and history of chronic pulmonary diseases, to explore potential effect modification. *P*-values for interaction were derived from likelihood ratio tests. Four sensitivity analyses were conducted to assess the robustness of the findings: excluding participants with <2 years of follow-up to minimize reverse causation; excluding participants with missing baseline covariates to evaluate the impact of multiple imputation; excluding participants on medications to reduce potential pharmacologic confounding; and applying a Fine–Gray competing risk model to account for competing events.

All analyses were performed using R software version 4.3.1 (R Foundation for Statistical Computing, Vienna, Austria), and a 2-sided *P*-value < .05 was considered statistically significant.

## 3. Results

### 3.1. Baseline characteristics

**Figure 1.**
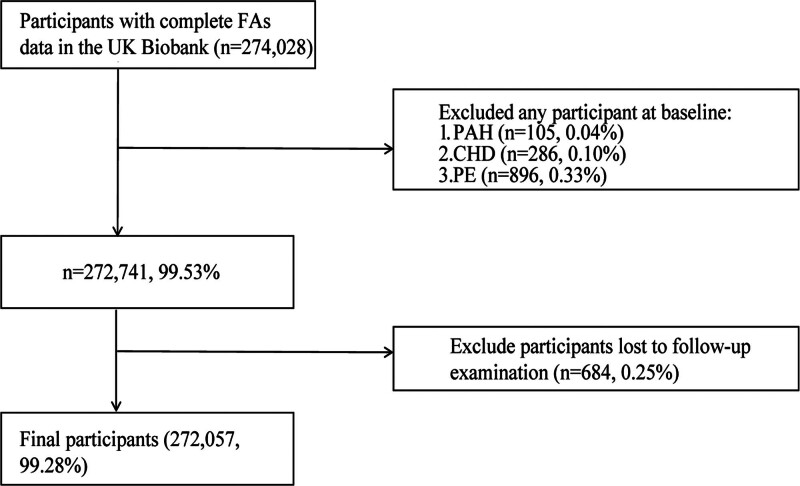
Flow diagram of participant selection.

Figure 1 illustrates the selection of study participants. Among the 272,057 individuals included in the final analysis, 1420 developed PAH during follow-up. Compared to non-PAH participants, those who developed PAH were older (62.04 ± 6.25 vs 56.54 ± 8.08 years), more frequently male (52.68% vs 45.96%), had a higher BMI (29.87 ± 6.42 vs 27.44 ± 4.77 kg/m^2^), and exhibited higher diet scores (5.19 ± 1.57 vs 5.06 ± 1.55) (all *P* < .01) (Table 1). Additionally, they had a higher prevalence of diabetes, hypertension, CVD, and were more likely to use lipid-lowering drugs, antihypertensives, and antidiabetic medications (all *P* < .001) (Table 1).

**Table 1:** Baseline demographic and clinical characteristics.

Characteristic	Total	Non-PAH	PAH	*P*-value
	(n = 272,057)	(n = 270,637)	(n = 1420)	
Age, years	56.56 ± 8.08	56.54 ± 8.08	62.04 ± 6.25	<.001
Male	125,140 (46.00%)	124,392 (45.96%)	748 (52.68%)	<.001
White	258,662 (95.08%)	257,323 (95.08%)	1339 (94.30%)	.17
Body mass index (kg/m^2^)	27.45 ± 4.78	27.44 ± 4.77	29.87 ± 6.42	<.001
Metabolic equivalent of task	2649.55 ± 2666.81	2650.26 ± 2666.15	2496.77 ± 2786.50	<.001
Townsend deprivation index	−1.36 ± 3.07	−1.36 ± 3.07	−0.46 ± 3.39	<.001
Diet score	5.06 ± 1.55	5.06 ± 1.55	5.19 ± 1.57	.008
Diabetes mellitus	16,650 (6.12%)	16,374 (6.05%)	276 (19.44%)	<.001
Hypertension	147,774 (54.32%)	146,666 (54.19%)	1108 (78.03%)	<.001
Cardiovascular diseases	12,050 (4.43%)	11,802 (4.36%)	248 (17.46%)	<.001
Chronic pulmonary diseases	1910 (0.70%)	1830 (0.68%)	80 (5.63%)	.894
Lipid-lowering drugs	48,208 (17.72%)	47,653 (17.61%)	555 (39.08%)	<.001
Antihypertensives	56,992 (20.95%)	56,291 (20.80%)	701 (49.37%)	<.001
Antidiabetic medications	10,162 (3.74%)	9980 (3.69%)	182 (12.82%)	<.001
Alcohol intake frequency				<.001
Never	21,630 (7.95%)	21,435 (7.92%)	195 (13.73%)	
Special occasions only	31,077 (11.42%)	30,885 (11.41%)	192 (13.52%)	
1 to 3 times a month	30,367 (11.16%)	30,214 (11.16%)	153 (10.77%)	
Once or twice a week	71,126 (26.14%)	70,790 (26.16%)	336 (23.66%)	
3 or 4 times a week	63,141 (23.21%)	62,891 (23.24%)	250 (17.61%)	
Daily or almost daily	54,716 (20.11%)	54,422 (20.11%)	294 (20.70%)	
Smoking status				<.001
Never	109,228 (40.15%)	108,782 (40.19%)	446 (31.41%)	
Previous	134,275 (49.36%)	133,507 (49.33%)	768 (54.08%)	
Current	28,554 (10.50%)	28,348 (10.47%)	206 (14.51%)	
DHA (mmol/l)	0.24 ± 0.08	0.24 ± 0.08	0.22 ± 0.08	<.001
LA (mmol/l)	3.46 ± 0.69	3.47 ± 0.69	3.24 ± 0.70	<.001
MUFA (mmol/l)	2.90 ± 0.84	2.90 ± 0.84	2.92 ± 0.87	.525
Omega.3 (mmol/l)	0.53 ± 0.22	0.53 ± 0.22	0.50 ± 0.22	<.001
Omega.6 (mmol/l)	4.51 ± 0.69	4.51 ± 0.69	4.30 ± 0.70	<.001
PUFA (mmol/l)	5.04 ± 0.81	5.04 ± 0.81	4.81 ± 0.82	<.001
SFA (mmol/l)	4.13 ± 0.97	4.13 ± 0.97	4.08 ± 1.02	.033
FA (mmol/l)	12.07 ± 2.44	12.07 ± 2.44	11.81 ± 2.51	<.001

Data were shown as mean (standard deviation) or frequency (percentage). DHA = docosahexaenoic acid, FA = total fatty acids, LA = linoleic acid, MUFA = monounsaturated fatty acids, Omega-3 = Omega-3 fatty acids, Omega-6 = Omega-6 fatty acids, PUFA = polyunsaturated fatty acids, SFA = saturated fatty acids.

Regarding circulating FAs, PAH cases had lower levels of DHA (0.22 ± 0.08 vs 0.24 ± 0.08 mmol/L), LA (3.24 ± 0.70 vs 3.47 ± 0.69 mmol/L), omega-3 (0.50 ± 0.22 vs 0.53 ± 0.22 mmol/L), omega-6 (4.30 ± 0.70 vs 4.51 ± 0.69 mmol/L), PUFA (4.81 ± 0.82 vs 5.04 ± 0.81 mmol/L), SFA (4.08 ± 1.02 vs 4.13 ± 0.97 mmol/L), and total FA (11.81 ± 2.51 vs 12.07 ± 2.44 mmol/L). MUFA levels were similar between groups (2.92 ± 0.87 vs 2.90 ± 0.84 mmol/L, *P* = .525) (Table 1).

### 3.2. Associations between fatty acids and PAH risk

Cox regression analyses revealed inverse associations between the 8 circulating FAs and the risk of PAH. In the fully adjusted model (Model 2), each 1-SD increase in DHA, LA, omega-3, omega-6, PUFA, and total FA was associated with an 11% to 19% lower risk of PAH (HR range: 0.81–0.86, all *P* < .001) (Table 2). Although MUFA showed no significant association in the age-, sex-, and race-adjusted model (Model 1), it was modestly inversely associated with PAH risk after full adjustment (HR = 0.92, 95% CI: 0.87–0.97, *P* < .001). Similarly, SFA also demonstrated an inverse association with PAH risk (HR = 0.92, 95% CI: 0.87–0.97, *P* = .003) (Table 2).

**Table 2:** Associations of fatty acids with risk of pulmonary arterial hypertension.

Types	Model 1		Model 2	
	HR (95% CI)	*P*	HR (95% CI)	*P*
DHA	0.70 (0.66–0.75)	<.001	0.84 (0.79–0.89)	<.001
LA	0.72 (0.68–0.76)	<.001	0.86 (0.81–0.91)	<.001
MUFA	0.99 (0.94–1.05)	.852	0.92 (0.87–0.97)	<.001
Omega-3	0.74 (0.7–0.79)	<.001	0.81 (0.77–0.87)	<.001
Omega-6	0.73 (0.69–0.77)	<.001	0.85 (0.8–0.9)	<.001
PUFA	0.70 (0.66–0.74)	<.001	0.83 (0.78–0.88)	<.001
SFA	0.92 (0.87–0.97)	.002	0.92 (0.87–0.97)	.003
FA	0.86 (0.81–0.91)	<.001	0.89 (0.84–0.94)	<.001

Hazard ratio (95% confidence interval) of per standard deviation increase of fatty acids. Model 1 was adjusted for age, sex, and race; Model 2 was further adjusted for additional covariates including the TDI  = Townsend Deprivation Index, BMI = body mass index, smoking status, DM = diabetes mellitus, hypertension, use of antihypertensive medication, lipid levels, antidiabetic medications, alcohol consumption, diet score, metabolic equivalent of task (MET), history of cardiovascular disease, CVD = chronic pulmonary diseases, DHA = docosahexaenoic acid, FA = total fatty acids, LA = linoleic acid, MUFA = monounsaturated fatty acids, Omega-3 = Omega-3 fatty acids, Omega-6 = Omega-6 fatty acids, PUFA = polyunsaturated fatty acids, SFA = saturated fatty acids.

### 3.3. Kaplan–Meier analysis of fatty acids and PAH risk

**Figure 2.**
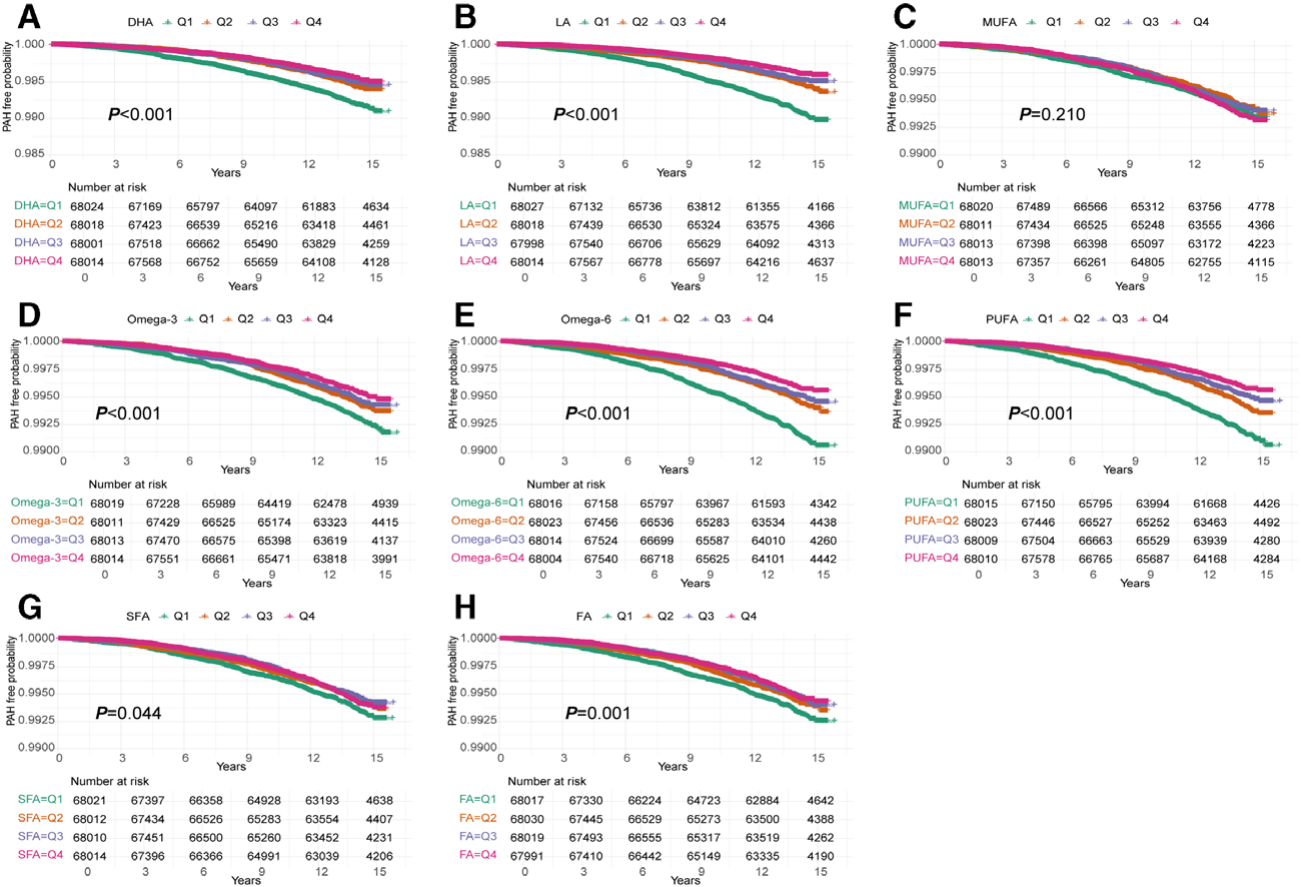
Kaplan–Meier curves showing the associations between quartiles of circulating fatty acids and the risk of pulmonary arterial hypertension. (A) docosahexaenoic acid (DHA); (B) linoleic acid (LA); (C) monounsaturated fatty acids (MUFA); (D) omega-3 fatty acids; (E) omega-6 fatty acids; (F) polyunsaturated fatty acids (PUFA); (G) saturated fatty acids (SFA); (H) total fatty acids (FA). Participants were stratified into quartiles (Q1–Q4), and differences were assessed using the log-rank test.

Kaplan–Meier curves indicated differences in PAH-free probability across quartiles of the 8 circulating FAs. Compared to the lowest quartile (Q1), higher quartiles of DHA, LA, omega-3 FAs, omega-6 FAs, PUFA, SFA, and total FA were consistently associated with a greater PAH-free probability throughout the follow-up period (all *P* < .05). The most pronounced separation was observed for DHA, LA, omega-3, omega-6, and PUFA (all *P* < .001) (Fig. 2). In contrast, MUFA showed no significant differences in PAH-free probability across quartiles (*P* = .210) (Fig. 2).

### 3.4. Complex dose–response relationships between FAs and PAH risk

**Figure 3.**
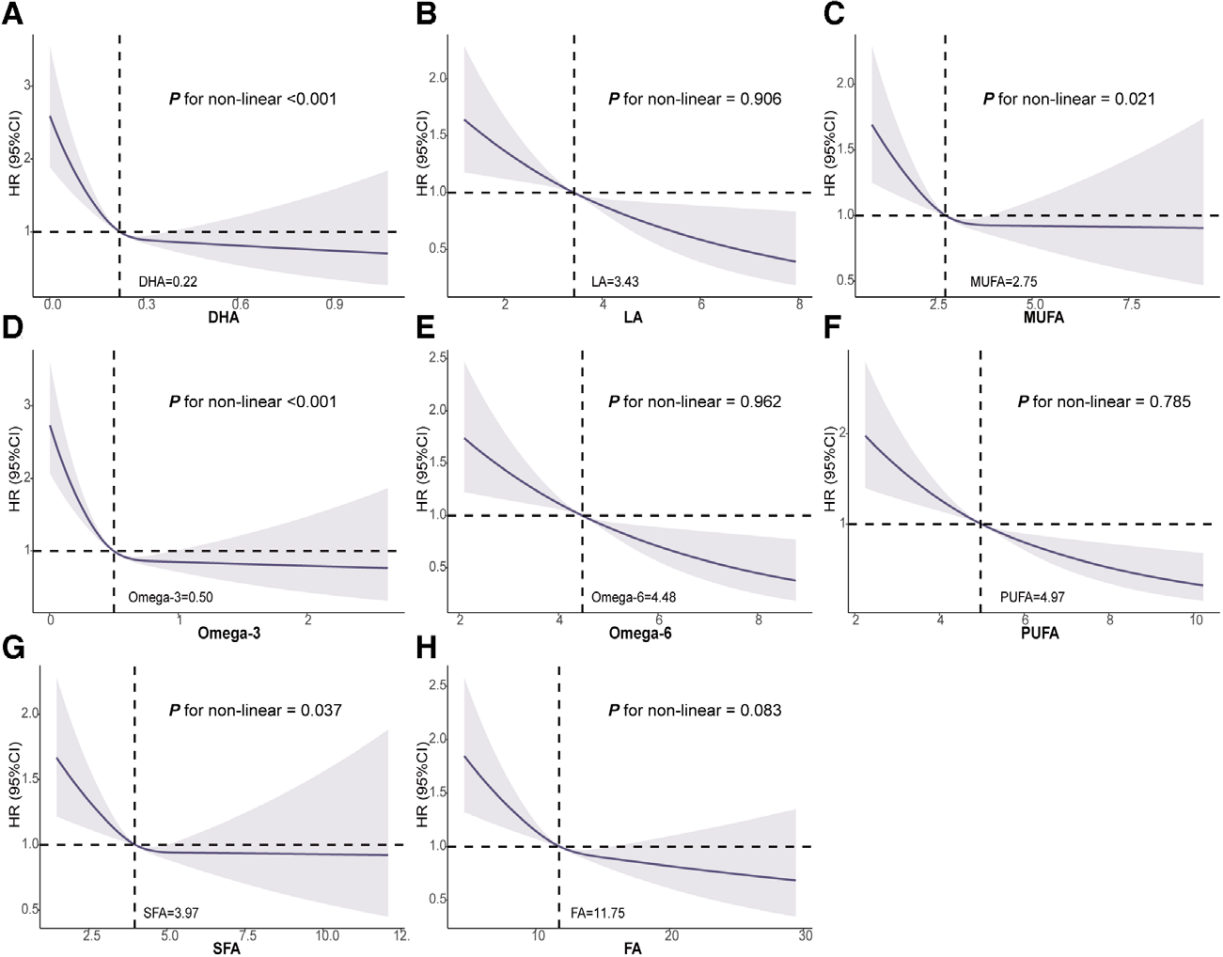
Association of the circulating fatty acids with o pulmonary arterial hypertension using RCS. RCS = restricted cubic spline. Models were fully adjusted for age, sex, race, Townsend Deprivation Index (TDI), body mass index (BMI), smoking status, diabetes mellitus (DM), hypertension, use of antihypertensive medication, lipid levels, antidiabetic medications, alcohol consumption, diet score, metabolic equivalent of task (MET), history of cardiovascular disease (CVD), and chronic pulmonary diseases. (A) docosahexaenoic acid (DHA); (B) linoleic acid (LA); (C) monounsaturated fatty acids (MUFA); (D) omega-3 fatty acids; (E) omega-6 fatty acids; (F) polyunsaturated fatty acids (PUFA); (G) saturated fatty acids (SFA); (H) total fatty acids (FA).

Restricted cubic spline analyses revealed nonlinear associations between DHA, MUFA, omega-3 FAs, and SFA concentrations and the risk of PAH. Specifically, concentrations of DHA < 0.22 mmol/L, MUFA < 2.75 mmol/L, omega-3 < 0.50 mmol/L, and SFA < 3.97 mmol/L were positively associated with PAH risk, with the risk decreasing progressively as concentrations increased. Beyond these thresholds, the associations reversed, with higher concentrations becoming protective against PAH, and risk curves plateauing at higher levels (Fig. 3). In contrast, LA, omega-6 FAs, PUFA, and total FA showed predominantly linear inverse associations with PAH risk (P for non-linearity > .05) (Fig. 3).

### 3.5. Accelerated failure time analysis

The accelerated failure time model results further confirmed that higher circulating FA concentrations were associated with a delayed onset of PAH. Compared to the lowest quartile (Q1), higher quartiles (Q2, Q3, and Q4) of DHA, LA, MUFA, omega-3, and omega-6 were linked to a substantial postponement of PAH onset, with the most significant effect observed in Q4, corresponding to a delay of approximately 60 to over 120 months (Figure S1, Supplemental Digital Content, https://links.lww.com/MD/R394). Similarly, Q3 and Q4 of PUFA, SFA, and total FA were associated with delayed onset, whereas Q2 showed no significant effect (Figure S1, Supplemental Digital Content, https://links.lww.com/MD/R394). Overall, progressively higher FA levels were linked to a later occurrence of PAH, with the strongest effects observed for omega-3, PUFA, and DHA, consistent with the findings from the Cox regression analyses.

### 3.6. Stratified analysis

Stratified analyses showed that the inverse associations between circulating FAs and PAH risk were generally consistent across demographic and clinical subgroups, though the magnitude of the associations varied (Tables S2–S11, Supplemental Digital Content, https://links.lww.com/MD/R394). In participants aged ≥ 60 years, females, Whites, individuals with BMI < 30 kg/m^2^, current smokers, current drinkers, those without diabetes, those with hypertension, and individuals without CVD or pulmonary disease, all 8 FAs were associated with a reduced risk of PAH (all *P* < .001). In contrast, among participants aged < 60 years, those with obesity (BMI ≥ 30 kg/m^2^), nonsmokers, nondrinkers, and those with CVD, only DHA and omega-3 FAs showed significant protective associations. In males and individuals with pulmonary disease, all 8 FAs, except for SFA, were significantly associated with reduced PAH risk. Conversely, in non-White and diabetic populations, none of the FAs demonstrated statistically significant associations with PAH risk (all *P* > .05).

Interaction analyses revealed significant effect modification by age on the associations of MUFA, SFA, and total FA with PAH risk. BMI significantly modified the associations of DHA, LA, omega-3, omega-6, and PUFA with PAH risk. Additionally, the effects of LA, omega-6, and PUFAs were significantly modified by smoking status, alcohol consumption, diabetes status, and CVD status (Tables S2–S11, Supplemental Digital Content, https://links.lww.com/MD/R394).

### 3.7. Sensitivity analyses

Sensitivity analyses confirmed the robustness of the primary findings, demonstrating consistent associations (Tables S12–S15, Supplemental Digital Content, https://links.lww.com/MD/R394). Specifically, excluding participants who developed PAH within the first 2 years of follow-up and applying the Fine–Gray competing risk model both showed that all 8 FAs remained inversely associated with PAH risk (Tables S12 and S14, Supplemental Digital Content, https://links.lww.com/MD/R394). When participants with missing baseline data were excluded, inverse associations persisted for DHA, LA, omega-3, omega-6, PUFA, and total FA, while associations for MUFA and SFA lost statistical significance (Table S13, Supplemental Digital Content, https://links.lww.com/MD/R394). Additionally, excluding individuals on medications at baseline revealed that, except for SFA, all other FAs maintained inverse associations with PAH risk (Table S15, Supplemental Digital Content, https://links.lww.com/MD/R394).

## 4. Discussion

This study observed inverse associations between multiple FAs, including DHA, LA, MUFA, omega-3, omega-6, PUFA, SFA, and total FA, and the risk of incident PAH. Subgroup analyses further revealed that these protective associations were most pronounced among older adults (≥60 years), women, individuals with BMI < 30 kg/m^2^, and current smokers and alcohol consumers.

The findings align with previous research demonstrating significant associations between various FAs and improved cardiopulmonary outcomes.^[21,22]^ Mozaffarian et al.^[23,24]^ reported that higher circulating levels of omega-3 PUFA were associated with reduced vascular inflammation, improved endothelial function, and lower rates of adverse cardiovascular events. However, Borges et al.,^[25]^ using genome-wide association study data, found no significant protective effects of PUFA, including omega-3 and omega-6, on CVD risk. This discrepancy may be attributed to pleiotropic effects related to lipoprotein-related traits. Additionally, a meta-analysis of randomized controlled trials suggested that supplementation with eicosapentaenoic acid and DHA reduced CVD risk in the general population, though findings were inconsistent in high-risk groups, especially in secondary prevention settings.^[26]^ Despite these inconsistencies, recent meta-analyses continue to support a dose-dependent benefit of long-chain omega-3 PUFAs.^[27,28]^ However, evidence from some studies raises concerns that prolonged high-dose supplementation could increase the risk of atrial fibrillation.^[29,30]^

Compared to CVD studies, research on PAH in relation to FAs is limited, with most studies focusing on surrogate endpoints such as pulmonary vascular resistance or right ventricular (RV) function. By leveraging population-based data, our study advances current knowledge by identifying associations between circulating FAs and PAH risk and highlighting the attenuation of these associations among individuals with diabetes and in non-White populations. These findings suggest potential population-specific pathophysiology and lipid metabolic heterogeneity. Mechanistically, omega-3 PUFAs have been proposed to exert preventive and therapeutic effects in PAH through anti-inflammatory actions, enhancement of the NO/sGC/cGMP signaling pathway, and inhibition of pro-inflammatory and pro-fibrotic mediators.^[31]^ Additionally, lipidomic analyses have shown that long-chain FA (LCFA) metabolic abnormalities can be detected prior to the clinical diagnosis of systemic sclerosis–associated PAH (SSc-PAH), demonstrating strong discriminatory performance and suggesting that FAs may serve as early biomarkers for PAH.^[32]^

Current evidence suggests that circulating FAs play a role in the initiation and progression of PAH through multiple pathways involving RV remodeling.^[33,34]^ Recent studies have demonstrated that pulmonary arterial smooth muscle cells and RV cardiomyocytes in PAH exhibit metabolic reprogramming, characterized by impaired FA oxidation (FAO) and a shift from glucose oxidation to a glycolytic phenotype, accompanied by lipid accumulation and mitochondrial dysfunction.^[35]^ Experimental data indicate that FAO enhances the sensitivity of microvascular endothelial transient receptor potential vanilloid 4 calcium channels, altering the rhythm and amplitude of calcium influx, thereby affecting endothelium-dependent vasodilation and barrier function.^[36]^ According to Piao et al.,^[37]^ impaired FAO in PAH may result from mitochondrial dysfunction, hypoxia-inducible factor activation, and inflammation-induced metabolic reprogramming, collectively shifting cardiomyocytes and pulmonary vascular cells toward glucose-dependent glycolysis. This shift reduces energy efficiency while promoting proliferative and anti-apoptotic phenotypes. Additionally, the role of PUFA in inflammation and vascular remodeling merits attention. Omega-3 PUFA may mitigate pulmonary vascular inflammation and endothelial dysfunction by inhibiting the NF-κB signaling pathway, reducing pro-inflammatory cytokine release (e.g., IL-6 and TNF-α), and modulating endothelial nitric oxide synthase activity.^[38]^ In contrast, SFA and omega-6 PUFA may exacerbate inflammation and promote aberrant pulmonary arterial smooth muscle cell proliferation by activating the Toll-like receptor 4–MyD88 signaling pathway.^[39]^ Moreover, lipid peroxidation products such as 4-hydroxynonenal can damage mitochondrial DNA and inhibit the activity of electron transport chain complexes, worsening FAO impairment and pulmonary vascular pathology.^[40,41]^

This study has several limitations that should be acknowledged. First, although the UKB provides a large, deeply phenotyped, and well-characterized cohort, its participants are predominantly of European ancestry, which may limit the generalizability of the findings to other ethnic groups or individuals with different socioeconomic or health backgrounds. Second, circulating FA levels in the UKB were measured at a single time point using NMR-based metabolomics, precluding the assessment of temporal variability and limiting our ability to evaluate longitudinal changes or within-person stability over time. Third, PAH incidence was determined through linkage to hospital admission data and mortality records coded by the ICD, which may be prone to misclassification or under-reporting, particularly during early or subclinical disease stages. The lack of uniform right heart catheterization data, the diagnostic gold standard, further limits diagnostic precision. Finally, while this study adjusted for a range of demographic, lifestyle, and clinical covariates, the possibility of residual or unmeasured confounding cannot be entirely excluded.

## 5. Conclusion

In conclusion, this large-scale, population-based cohort study demonstrates consistent inverse associations between higher circulating levels of multiple FAs and the risk of incident PAH, with these associations being confirmed through extensive sensitivity analyses. These findings emphasize the complex interplay between systemic lipid metabolism and pulmonary vascular pathophysiology. Importantly, the results extend the cardioprotective role of FAs to the pulmonary vasculature, offering novel, population-level evidence that systemic FA profiles may influence PAH risk. Furthermore, these findings position circulating FAs as potential biomarkers and therapeutic targets, with significant implications for primary prevention and adjunctive management strategies in PAH.

## Acknowledgments

We gratefully acknowledge the commitment and dedication of the participants of the UK Biobank.

## Author contributions

**Writing – original draft:** Shuo Liu.

**Writing – review & editing:** Shuo Liu.

**Validation:** Haobei Yuan.

**Software:** Jingxian Cao, Liang Cai.

**Investigation:** Quanlei Wang.

**Supervision:** Xiaoping Zhou.

## Supplementary Material


