# Supplementary material for: Circulating fatty acid profiles and risk of pulmonary arterial hypertension: Evidence from a large prospective cohort study
Source: Medicine (Baltimore). 2026 Feb 28;105(9):e47645. doi: 10.1097/MD.0000000000047645 (PMC12956177; doi:10.1097/MD.0000000000047645)
Supplement: Supplementary file 1 [file medi-105-e47645-s001.docx]

**Circulating Fatty Acid Profiles and Risk of Pulmonary Arterial Hypertension: Evidence from a Large Prospective Cohort Study**

[Table S1. Information on missing covariates among 272,057 UK Biobank participants.](#_Toc201022280)

[Table S2. Subgroup analyses of the associations between circulating fatty acid levels and pulmonary arterial hypertension, stratified by baseline age.](#_Toc201022281)

[Table S3. Subgroup analyses of the associations between circulating fatty acid levels and pulmonary arterial hypertension, stratified by baseline sex status.](#_Toc201022282)

[Table S4. Subgroup analyses of the associations between circulating fatty acid levels and pulmonary arterial hypertension, stratified by baseline race status.](#_Toc201022283)

[Table S5. Subgroup analyses of the associations between circulating fatty acid levels and pulmonary arterial hypertension, stratified by baseline body mass index.](#_Toc201022284)

[Table S6. Subgroup analyses of the associations between circulating fatty acid levels and pulmonary arterial hypertension, stratified by baseline smoking status.](#_Toc201022285)

[Table S7. Subgroup analyses of the associations between circulating fatty acid levels and pulmonary arterial hypertension, stratified by baseline alcohol status.](#_Toc201022286)

[Table S8. Subgroup analyses of the associations between circulating fatty acid levels and pulmonary arterial hypertension, stratified by baseline diabetes mellitus status.](#_Toc201022287)

[Table S9. Subgroup analyses of the associations between circulating fatty acid levels and pulmonary arterial hypertension, stratified by baseline hypertension status.](#_Toc201022288)

[Table S10. Subgroup analyses of the associations between circulating fatty acid levels and pulmonary arterial hypertension, stratified by baseline CVD status.](#_Toc201022289)

[Table S11. Subgroup analyses of the associations between circulating fatty acid levels and pulmonary arterial hypertension, stratified by baseline chronic pulmonary diseases status.](#_Toc201022290)

[Table S12. Associations of fatty acids with risk of pulmonary arterial hypertension after excluding participants within two years of follow-up.](#_Toc201022291)

[Table S13. Associations of fatty acids with risk of pulmonary arterial hypertension after excluding participants with any missing value at baseline.](#_Toc201022292)

[Table S14. Associations of fatty acids with risk of pulmonary arterial hypertension after excluding participants with Fine-Gray competing risk model.](#_Toc201022293)

[Table S15. Associations of fatty acids with risk of pulmonary arterial hypertension after excluding participants with any drugs at baseline.](#_Toc201022294)

[Figure S1. Adjusted median time differences for pulmonary arterial hypertension across fatty acid quartiles.](#_Toc201022295)

| Table S1. Information on missing covariates among 272,057 UK Biobank participants. | | |
| --- | --- | --- |
| **Covariates** | **Number of missing** | **Missing rates (%)** |
| Race and ethnicity | 1215 | 0.45 |
| Townsend Deprivation Index | 326 | 0.12 |
| Body mass index | 1,013 | 0.37 |
| Antihypertensives | 248 | 0.09 |
| Lowering lipids drugs | 248 | 0.09 |
| Antidiabetic medications | 105 | 0.04 |
| Smoking status | 1323 | 0.49 |
| Alcohol intake frequency | 548 | 0.2 |
| Diet score | 10104 | 3.71 |
| Metabolic equivalent of task | 62023 | 22.8 |
| C-reactive protein | 12,464 | 4.58 |

| Table S2. Subgroup analyses of the associations between circulating fatty acid levels and pulmonary arterial hypertension, stratified by baseline age. | | | | | |
| --- | --- | --- | --- | --- | --- |
| **Types** | **< 60 years** | | **≥ 60 years** | | ***P* for interaction** |
|  | **HR (95% CI)** | ***P*** | **HR (95% CI)** | ***P*** |  |
| DHA | 0.81 (0.70–0.92) | 0.002 | 0.85 (0.79–0.91) | <0.001 | 0.096 |
| LA | 0.98 (0.88–1.09) | 0.712 | 0.81 (0.76–0.87) | <0.001 | 0.428 |
| MUFA | 1.04 (0.95–1.14) | 0.401 | 0.86 (0.81–0.92) | <0.001 | 0.000 |
| Omega-3 | 0.83 (0.73–0.94) | 0.004 | 0.81 (0.75–0.87) | <0.001 | 0.990 |
| Omega-6 | 0.96 (0.86–1.07) | 0.507 | 0.81 (0.75–0.86) | <0.001 | 0.392 |
| PUFA | 0.93 (0.83–1.04) | 0.188 | 0.78 (0.73–0.84) | <0.001 | 0.412 |
| SFA | 1.07 (0.98–1.18) | 0.151 | 0.86 (0.80–0.92) | <0.001 | 0.000 |
| FA | 1.02 (0.93–1.13) | 0.640 | 0.83 (0.78–0.89) | <0.001 | 0.002 |

Hazard ratio (95% confidence interval) of per standard deviation increase of fatty acids. Models were fully adjusted for age, sex, race, Townsend Deprivation Index (TDI), body mass index (BMI), smoking status, diabetes mellitus (DM), hypertension, use of antihypertensive medication, lipid levels, antidiabetic medications, alcohol consumption, diet score, metabolic equivalent of task (MET), history of cardiovascular disease (CVD), and chronic pulmonary diseases. DHA: docosahexaenoic acid; LA: linoleic acid; MUFA: monounsaturated fatty acids; Omega-3: Omega-3 fatty acids; Omega-6: Omega-6 fatty acids; PUFA: polyunsaturated fatty acids; SFA: saturated fatty acids; FA: total fatty acids.

| Table S3. Subgroup analyses of the associations between circulating fatty acid levels and pulmonary arterial hypertension, stratified by baseline sex status. | | | | | |
| --- | --- | --- | --- | --- | --- |
| **Types** | **Female** | | **Male** | | ***P* for interaction** |
|  | **HR (95% CI)** | ***P*** | **HR (95% CI)** | ***P*** |  |
| DHA | 0.87 (0.80–0.95) | 0.002 | 0.78 (0.71–0.86) | <0.001 | 0.095 |
| LA | 0.82 (0.76–0.90) | <0.001 | 0.89 (0.82–0.97) | 0.005 | 0.106 |
| MUFA | 0.89 (0.82–0.97) | 0.009 | 0.93 (0.86–1.00) | 0.045 | 0.304 |
| Omega-3 | 0.85 (0.78–0.92) | <0.001 | 0.76 (0.69–0.83) | <0.001 | 0.071 |
| Omega-6 | 0.82 (0.75–0.89) | <0.001 | 0.87 (0.80–0.94) | 0.001 | 0.151 |
| PUFA | 0.81 (0.74–0.88) | <0.001 | 0.83 (0.76–0.91) | <0.001 | 0.404 |
| SFA | 0.90 (0.82–0.97) | 0.010 | 0.93 (0.86–1.00) | 0.056 | 0.232 |
| FA | 0.86 (0.79–0.94) | <0.001 | 0.90 (0.83–0.97) | 0.005 | 0.231 |

Hazard ratio (95% confidence interval) of per standard deviation increase of fatty acids. Models were fully adjusted for age, race, Townsend Deprivation Index (TDI), body mass index (BMI), smoking status, diabetes mellitus (DM), hypertension, use of antihypertensive medication, lipid levels, antidiabetic medications, alcohol consumption, diet score, metabolic equivalent of task (MET), history of cardiovascular disease (CVD), and chronic pulmonary diseases. DHA: docosahexaenoic acid; LA: linoleic acid; MUFA: monounsaturated fatty acids; Omega-3: Omega-3 fatty acids; Omega-6: Omega-6 fatty acids; PUFA: polyunsaturated fatty acids; SFA: saturated fatty acids; FA: total fatty acids.

| Table S4. Subgroup analyses of the associations between circulating fatty acid levels and pulmonary arterial hypertension, stratified by baseline race status. | | | | | |
| --- | --- | --- | --- | --- | --- |
| **Types** | **Non-white** | | **White** | | ***P* for interaction** |
|  | **HR (95% CI)** | ***P*** | **HR (95% CI)** | ***P*** |  |
| DHA | 0.88 (0.71–1.11) | 0.278 | 0.82 (0.77–0.88) | <0.001 | 0.392 |
| LA | 0.95 (0.78–1.17) | 0.647 | 0.85 (0.80–0.91) | <0.001 | 0.555 |
| MUFA | 0.97 (0.77–1.23) | 0.829 | 0.92 (0.87–0.97) | 0.003 | 0.806 |
| Omega-3 | 0.84 (0.67–1.06) | 0.143 | 0.81 (0.76–0.86) | <0.001 | 0.555 |
| Omega-6 | 0.92 (0.74–1.14) | 0.447 | 0.85 (0.79–0.90) | <0.001 | 0.646 |
| PUFA | 0.88 (0.71–1.11) | 0.281 | 0.82 (0.77–0.87) | <0.001 | 0.657 |
| SFA | 0.94 (0.74–1.18) | 0.580 | 0.92 (0.87–0.98) | 0.006 | 0.929 |
| FA | 0.93 (0.74–1.16) | 0.513 | 0.89 (0.84–0.94) | <0.001 | 0.865 |

Hazard ratio (95% confidence interval) of per standard deviation increase of fatty acids. Models were fully adjusted for age, sex, Townsend Deprivation Index (TDI), body mass index (BMI), smoking status, diabetes mellitus (DM), hypertension, use of antihypertensive medication, lipid levels, antidiabetic medications, alcohol consumption, diet score, metabolic equivalent of task (MET), history of cardiovascular disease (CVD), and chronic pulmonary diseases. DHA: docosahexaenoic acid; LA: linoleic acid; MUFA: monounsaturated fatty acids; Omega-3: Omega-3 fatty acids; Omega-6: Omega-6 fatty acids; PUFA: polyunsaturated fatty acids; SFA: saturated fatty acids; FA: total fatty acids.

| Table S5. Subgroup analyses of the associations between circulating fatty acid levels and pulmonary arterial hypertension, stratified by baseline body mass index. | | | | | |
| --- | --- | --- | --- | --- | --- |
| **Types** | **< 30 kg/m^2^** | | **≥ 30 kg/m^2^** | | ***P* for interaction** |
|  | **HR (95% CI)** | ***P*** | **HR (95% CI)** | ***P*** |  |
| DHA | 0.78 (0.72–0.85) | <0.001 | 0.89 (0.81–0.98) | 0.016 | 0.018 |
| LA | 0.82 (0.75–0.88) | <0.001 | 0.92 (0.84–1.01) | 0.062 | 0.034 |
| MUFA | 0.91 (0.84–0.98) | 0.012 | 0.96 (0.89–1.03) | 0.246 | 0.318 |
| Omega-3 | 0.76 (0.70–0.82) | <0.001 | 0.88 (0.81–0.97) | 0.009 | 0.007 |
| Omega-6 | 0.88 (0.74–0.87) | <0.001 | 0.91 (0.84–1.01) | 0.053 | 0.018 |
| PUFA | 0.77 (0.71–0.83) | <0.001 | 0.90 (0.82–0.98) | 0.019 | 0.005 |
| SFA | 0.90 (0.83–0.97) | 0.007 | 0.96 (0.89–1.04) | 0.289 | 0.175 |
| FA | 0.85 (0.79–0.92) | <0.001 | 0.94 (0.87–1.02) | 0.123 | 0.051 |

Hazard ratio (95% confidence interval) of per standard deviation increase of fatty acids. Models were fully adjusted for age, sex, race, Townsend Deprivation Index (TDI), body mass index (BMI), smoking status, diabetes mellitus (DM), hypertension, use of antihypertensive medication, lipid levels, antidiabetic medications, alcohol consumption, diet score, metabolic equivalent of task (MET), history of cardiovascular disease (CVD), and chronic pulmonary diseases. DHA: docosahexaenoic acid; LA: linoleic acid; MUFA: monounsaturated fatty acids; Omega-3: Omega-3 fatty acids; Omega-6: Omega-6 fatty acids; PUFA: polyunsaturated fatty acids; SFA: saturated fatty acids; FA: total fatty acids.

| Table S6. Subgroup analyses of the associations between circulating fatty acid levels and pulmonary arterial hypertension, stratified by baseline smoking status. | | | | | |
| --- | --- | --- | --- | --- | --- |
| **Types** | **Not active** | | **Active** | | ***P* for interaction** |
|  | **HR (95% CI)** | ***P*** | **HR (95% CI)** | ***P*** |  |
| DHA | 0.85 (0.77–0.95) | 0.004 | 0.82 (0.76–0.89) | <0.001 | 0.697 |
| LA | 0.98 (0.89–1.09) | 0.741 | 0.88 (0.75–0.87) | <0.001 | 0.026 |
| MUFA | 0.93 (0.84–1.03) | 0.154 | 0.92 (0.86–0.98) | 0.009 | 0.857 |
| Omega-3 | 0.82 (0.74–0.92) | 0.000 | 0.80 (0.75–0.87) | <0.001 | 0.674 |
| Omega-6 | 0.98 (0.88–1.08) | 0.629 | 0.80 (0.74–0.86) | <0.001 | 0.020 |
| PUFA | 0.93 (0.84–1.03) | 0.158 | 0.78 (0.72–0.84) | <0.001 | 0.039 |
| SFA | 0.95 (0.86–1.05) | 0.295 | 0.91 (0.85–0.97) | 0.006 | 0.664 |
| FA | 0.93 (0.84–1.03) | 0.173 | 0.87 (0.81–0.93) | <0.001 | 0.371 |

Hazard ratio (95% confidence interval) of per standard deviation increase of fatty acids. Models were fully adjusted for age, sex, race, Townsend Deprivation Index (TDI), body mass index (BMI), diabetes mellitus (DM), hypertension, use of antihypertensive medication, lipid levels, antidiabetic medications, alcohol consumption, diet score, metabolic equivalent of task (MET), history of cardiovascular disease (CVD), and chronic pulmonary diseases. DHA: docosahexaenoic acid; LA: linoleic acid; MUFA: monounsaturated fatty acids; Omega-3: Omega-3 fatty acids; Omega-6: Omega-6 fatty acids; PUFA: polyunsaturated fatty acids; SFA: saturated fatty acids; FA: total fatty acids.

| Table S7. Subgroup analyses of the associations between circulating fatty acid levels and pulmonary arterial hypertension, stratified by baseline alcohol status. | | | | | |
| --- | --- | --- | --- | --- | --- |
| **Types** | **Not active** | | **Active** | | ***P* for interaction** |
|  | **HR (95% CI)** | ***P*** | **HR (95% CI)** | ***P*** |  |
| DHA | 0.84 (0.76–0.93) | 0.001 | 0.82 (0.76–0.89) | <0.001 | 0.370 |
| LA | 0.94 (0.86–1.03) | 0.219 | 0.80 (0.74–0.87) | <0.001 | 0.001 |
| MUFA | 0.96 (0.88–1.05) | 0.359 | 0.89 (0.83–0.96) | 0.002 | 0.203 |
| Omega-3 | 0.82 (0.75–0.91) | 0.000 | 0.80 (0.74–0.87) | <0.001 | 0.452 |
| Omega-6 | 0.93 (0.85–1.02) | 0.146 | 0.80 (0.74–0.86) | <0.001 | 0.001 |
| PUFA | 0.90 (0.82–0.98) | 0.022 | 0.78 (0.72–0.84) | <0.001 | 0.002 |
| SFA | 0.95 (0.87–1.04) | 0.248 | 0.90 (0.84–0.97) | 0.004 | 0.207 |
| FA | 0.93 (0.86–1.02) | 0.131 | 0.85 (0.80–0.92) | <0.001 | 0.049 |

Hazard ratio (95% confidence interval) of per standard deviation increase of fatty acids. Models were fully adjusted for age, sex, race, Townsend Deprivation Index (TDI), body mass index (BMI), smoking status, diabetes mellitus (DM), hypertension, use of antihypertensive medication, lipid levels, antidiabetic medications, diet score, metabolic equivalent of task (MET), history of cardiovascular disease (CVD), and chronic pulmonary diseases. DHA: docosahexaenoic acid; LA: linoleic acid; MUFA: monounsaturated fatty acids; Omega-3: Omega-3 fatty acids; Omega-6: Omega-6 fatty acids; PUFA: polyunsaturated fatty acids; SFA: saturated fatty acids; FA: total fatty acids.

| Table S8. Subgroup analyses of the associations between circulating fatty acid levels and pulmonary arterial hypertension, stratified by baseline diabetes mellitus status. | | | | | |
| --- | --- | --- | --- | --- | --- |
| **Types** | **No** | | **Yes** | | ***P* for interaction** |
|  | **HR (95% CI)** | ***P*** | **HR (95% CI)** | ***P*** |  |
| DHA | 0.81 (0.75–0.87) | <0.001 | 0.94 (0.81–1.08) | 0.362 | 0.116 |
| LA | 0.83 (0.77–0.88) | <0.001 | 1.01 (0.88–1.15) | 0.889 | 0.008 |
| MUFA | 0.91 (0.85–0.97) | 0.002 | 0.96 (0.86–1.06) | 0.418 | 0.234 |
| Omega-3 | 0.79 (0.74–0.84) | <0.001 | 0.90 (0.79–1.04) | 0.143 | 0.090 |
| Omega-6 | 0.82 (0.77–0.88) | <0.001 | 0.97 (0.85–1.11) | 0.680 | 0.022 |
| PUFA | 0.79 (0.74–0.85) | <0.001 | 0.95 (0.83–1.09) | 0.452 | 0.015 |
| SFA | 0.91 (0.86–0.97) | 0.005 | 0.94 (0.84–1.05) | 0.299 | 0.435 |
| FA | 0.87 (0.81–0.92) | <0.001 | 0.95 (0.85–1.06) | 0.349 | 0.110 |

Hazard ratio (95% confidence interval) of per standard deviation increase of fatty acids. Models were fully adjusted for age, sex, race, Townsend Deprivation Index (TDI), body mass index (BMI), smoking status, hypertension, use of antihypertensive medication, lipid levels, antidiabetic medications, alcohol consumption, diet score, metabolic equivalent of task (MET), history of cardiovascular disease (CVD), and chronic pulmonary diseases. DHA: docosahexaenoic acid; LA: linoleic acid; MUFA: monounsaturated fatty acids; Omega-3: Omega-3 fatty acids; Omega-6: Omega-6 fatty acids; PUFA: polyunsaturated fatty acids; SFA: saturated fatty acids; FA: total fatty acids.

| Table S9. Subgroup analyses of the associations between circulating fatty acid levels and pulmonary arterial hypertension, stratified by baseline hypertension status. | | | | | |
| --- | --- | --- | --- | --- | --- |
| **Types** | **No** | | **Yes** | | ***P* for interaction** |
|  | **HR (95% CI)** | ***P*** | **HR (95% CI)** | ***P*** |  |
| DHA | 0.78 (0.68–0.89) | <0.001 | 0.85 (0.79–0.91) | <0.001 | 0.131 |
| LA | 0.82 (0.72–0.92) | 0.001 | 0.87 (0.81–0.93) | <0.001 | 0.251 |
| MUFA | 1.01 (0.89–1.14) | 0.898 | 0.90 (0.85–0.96) | 0.001 | 0.230 |
| Omega-3 | 0.80 (0.70–0.91) | 0.001 | 0.81 (0.76–0.87) | <0.001 | 0.358 |
| Omega-6 | 0.81 (0.72–0.92) | <0.001 | 0.86 (0.80–0.92) | <0.001 | 0.235 |
| PUFA | 0.79 (0.69–0.89) | <0.001 | 0.83 (0.78–0.89) | <0.001 | 0.203 |
| SFA | 0.98 (0.87–1.11) | 0.731 | 0.91 (0.85–0.97) | 0.002 | 0.549 |
| FA | 0.92 (0.81–1.04) | 0.177 | 0.88 (0.82–0.93) | <0.001 | 0.882 |

Hazard ratio (95% confidence interval) of per standard deviation increase of fatty acids. Models were fully adjusted for age, sex, race, Townsend Deprivation Index (TDI), body mass index (BMI), smoking status, diabetes mellitus (DM), use of antihypertensive medication, lipid levels, antidiabetic medications, alcohol consumption, diet score, metabolic equivalent of task (MET), history of cardiovascular disease (CVD), and chronic pulmonary diseases. DHA: docosahexaenoic acid; LA: linoleic acid; MUFA: monounsaturated fatty acids; Omega-3: Omega-3 fatty acids; Omega-6: Omega-6 fatty acids; PUFA: polyunsaturated fatty acids; SFA: saturated fatty acids; FA: total fatty acids.

| Table S10. Subgroup analyses of the associations between circulating fatty acid levels and pulmonary arterial hypertension, stratified by baseline CVD status. | | | | | |
| --- | --- | --- | --- | --- | --- |
| **Types** | **No** | | **Yes** | | ***P* for interaction** |
|  | **HR (95% CI)** | ***P*** | **HR (95% CI)** | ***P*** |  |
| DHA | 0.83 (0.77–0.89) | <0.001 | 0.85 (0.72–0.99) | 0.036 | 0.147 |
| LA | 0.85 (0.80–0.91) | <0.001 | 0.89 (0.77–1.04) | 0.138 | 0.016 |
| MUFA | 0.92 (0.86–0.98) | 0.006 | 0.91 (0.80–1.03) | 0.139 | 0.872 |
| Omega-3 | 0.81 (0.76–0.87) | <0.001 | 0.79 (0.68–0.93) | 0.004 | 0.569 |
| Omega-6 | 0.84 (0.79–0.90) | <0.001 | 0.89 (0.76–1.03) | 0.104 | 0.018 |
| PUFA | 0.82 (0.77–0.87) | <0.001 | 0.85 (0.73–0.98) | 0.03 | 0.028 |
| SFA | 0.92 (0.87–0.98) | 0.009 | 0.90 (0.79–1.03) | 0.131 | 0.751 |
| FA | 0.88 (0.83–0.94) | <0.001 | 0.89 (0.77–1.01) | 0.073 | 0.412 |

Hazard ratio (95% confidence interval) of per standard deviation increase of fatty acids. Models were fully adjusted for age, sex, race, Townsend Deprivation Index (TDI), body mass index (BMI), smoking status, diabetes mellitus (DM), hypertension, use of antihypertensive medication, lipid levels, antidiabetic medications, alcohol consumption, diet score, metabolic equivalent of task (MET) and chronic pulmonary diseases. DHA: docosahexaenoic acid; LA: linoleic acid; MUFA: monounsaturated fatty acids; Omega-3: Omega-3 fatty acids; Omega-6: Omega-6 fatty acids; PUFA: polyunsaturated fatty acids; SFA: saturated fatty acids; FA: total fatty acids.

| Table S11. Subgroup analyses of the associations between circulating fatty acid levels and pulmonary arterial hypertension, stratified by baseline chronic pulmonary diseases status. | | | | | |
| --- | --- | --- | --- | --- | --- |
| **Types** | **No** | | **Yes** | | ***P* for interaction** |
|  | **HR (95% CI)** | ***P*** | **HR (95% CI)** | ***P*** |  |
| DHA | 0.84 (0.79–0.89) | <0.001 | 0.74 (0.55–1.00) | 0.049 | 0.616 |
| LA | 0.87 (0.82–0.93) | <0.001 | 0.67 (0.51–0.87) | 0.003 | 0.454 |
| MUFA | 0.93 (0.88–0.98) | 0.007 | 0.77 (0.60–0.99) | 0.039 | 0.298 |
| Omega-3 | 0.82 (0.77–0.87) | <0.001 | 0.67 (0.50–0.90) | 0.008 | 0.546 |
| Omega-6 | 0.86 (0.81–0.92) | <0.001 | 0.68 (0.52–0.88) | 0.004 | 0.393 |
| PUFA | 0.83 (0.79–0.89) | <0.001 | 0.65 (0.50–0.85) | 0.002 | 0.545 |
| SFA | 0.93 (0.87–0.98) | 0.007 | 0.84 (0.66–1.07) | 0.162 | 0.724 |
| FA | 0.89 (0.84–0.95) | <0.001 | 0.75 (0.58–0.96) | 0.023 | 0.994 |

Hazard ratio (95% confidence interval) of per standard deviation increase of fatty acids. Models were fully adjusted for age, sex, race, Townsend Deprivation Index (TDI), body mass index (BMI), smoking status, diabetes mellitus (DM), hypertension, use of antihypertensive medication, lipid levels, antidiabetic medications, alcohol consumption, diet score, metabolic equivalent of task (MET) and history of cardiovascular disease (CVD). DHA: docosahexaenoic acid; LA: linoleic acid; MUFA: monounsaturated fatty acids; Omega-3: Omega-3 fatty acids; Omega-6: Omega-6 fatty acids; PUFA: polyunsaturated fatty acids; SFA: saturated fatty acids; FA: total fatty acids.

| Table S12. Associations of fatty acids with risk of pulmonary arterial hypertension after excluding participants within two years of follow-up. | | |
| --- | --- | --- |
| **Types** | **HR (95%CI)** | ***P*** |
| DHA | 0.85 (0.79-0.90) | <0.001 |
| LA | 0.87 (0.82-0.92) | <0.001 |
| MUFA | 0.92 (0.87-0.98) | 0.004 |
| Omega-3 | 0.82 (0.78-0.88) | <0.001 |
| Omega-6 | 0.86 (0.81-0.91) | <0.001 |
| PUFA | 0.84 (0.79-0.89) | <0.001 |
| SFA | 0.93 (0.88-0.98) | 0.012 |
| FA | 0.90 (0.85-0.95) | <0.001 |

Hazard ratio (95% confidence interval) of per standard deviation increase of fatty acids. Models were fully adjusted for age, sex, race, Townsend Deprivation Index (TDI), body mass index (BMI), smoking status, diabetes mellitus (DM), hypertension, alcohol consumption, diet score, metabolic equivalent of task (MET), history of cardiovascular disease (CVD), and chronic pulmonary diseases. DHA: docosahexaenoic acid; LA: linoleic acid; MUFA: monounsaturated fatty acids; Omega-3: Omega-3 fatty acids; Omega-6: Omega-6 fatty acids; PUFA: polyunsaturated fatty acids; SFA: saturated fatty acids; FA: total fatty acids.

| Table S13. Associations of fatty acids with risk of pulmonary arterial hypertension after excluding participants with any missing value at baseline. | | |
| --- | --- | --- |
| **Types** | **HR (95%CI)** | ***P*** |
| DHA | 0.86 (0.80-0.93) | <0.001 |
| LA | 0.87 (0.81-0.94) | <0.001 |
| MUFA | 0.95 (0.89-1.02) | 0.132 |
| Omega-3 | 0.86 (0.80-0.93) | <0.001 |
| Omega-6 | 0.87 (0.81-0.93) | <0.001 |
| PUFA | 0.85 (0.79-0.91) | <0.001 |
| SFA | 0.95 (0.89-1.02) | 0.134 |
| FA | 0.92 (0.86-0.98) | 0.012 |

Hazard ratio (95% confidence interval) of per standard deviation increase of fatty acids. Models were fully adjusted for age, sex, race, Townsend Deprivation Index (TDI), body mass index (BMI), smoking status, diabetes mellitus (DM), hypertension, alcohol consumption, diet score, metabolic equivalent of task (MET), history of cardiovascular disease (CVD), and chronic pulmonary diseases. DHA: docosahexaenoic acid; LA: linoleic acid; MUFA: monounsaturated fatty acids; Omega-3: Omega-3 fatty acids; Omega-6: Omega-6 fatty acids; PUFA: polyunsaturated fatty acids; SFA: saturated fatty acids; FA: total fatty acids.

| Table S14. Associations of fatty acids with risk of pulmonary arterial hypertension after excluding participants with Fine-Gray competing risk model. | | |
| --- | --- | --- |
| **Types** | **HR (95%CI)** | ***P*** |
| DHA | 0.85 (0.79-0.91) | <0.001 |
| LA | 0.87 (0.82-0.93) | <0.001 |
| MUFA | 0.92 (0.87-0.97) | 0.003 |
| Omega-3 | 0.83 (0.77-0.88) | <0.001 |
| Omega-6 | 0.86 (0.81-0.92) | <0.001 |
| PUFA | 0.84 (0.79-0.89) | <0.001 |
| SFA | 0.92 (0.87-0.98) | 0.008 |
| FA | 0.89 (0.84-0.95) | <0.001 |

Hazard ratio (95% confidence interval) of per standard deviation increase of fatty acids. Models were fully adjusted for age, sex, race, Townsend Deprivation Index (TDI), body mass index (BMI), smoking status, diabetes mellitus (DM), hypertension, alcohol consumption, diet score, metabolic equivalent of task (MET), history of cardiovascular disease (CVD), and chronic pulmonary diseases. DHA: docosahexaenoic acid; LA: linoleic acid; MUFA: monounsaturated fatty acids; Omega-3: Omega-3 fatty acids; Omega-6: Omega-6 fatty acids; PUFA: polyunsaturated fatty acids; SFA: saturated fatty acids; FA: total fatty acids.

| Table S15. Associations of fatty acids with risk of pulmonary arterial hypertension after excluding participants with any drugs at baseline. | | |
| --- | --- | --- |
| **Types** | **HR (95%CI)** | ***P*** |
| DHA | 0.82 (0.74-0.90) | <0.001 |
| LA | 0.82 (0.75-0.89) | <0.001 |
| MUFA | 0.91 (0.84-1.00) | 0.044 |
| Omega-3 | 0.81 (0.74-0.89) | <0.001 |
| Omega-6 | 0.81 (0.74-0.88) | <0.001 |
| PUFA | 0.79 (0.72-0.86) | <0.001 |
| SFA | 0.92 (0.85-1.00) | 0.065 |
| FA | 0.87 (0.80-0.95) | 0.002 |

Hazard ratio (95% confidence interval) of per standard deviation increase of fatty acids. Models were fully adjusted for age, sex, race, Townsend Deprivation Index (TDI), body mass index (BMI), smoking status, diabetes mellitus (DM), hypertension, alcohol consumption, diet score, metabolic equivalent of task (MET), history of cardiovascular disease (CVD), and chronic pulmonary diseases. DHA: docosahexaenoic acid; LA: linoleic acid; MUFA: monounsaturated fatty acids; Omega-3: Omega-3 fatty acids; Omega-6: Omega-6 fatty acids; PUFA: polyunsaturated fatty acids; SFA: saturated fatty acids; FA: total fatty acids.


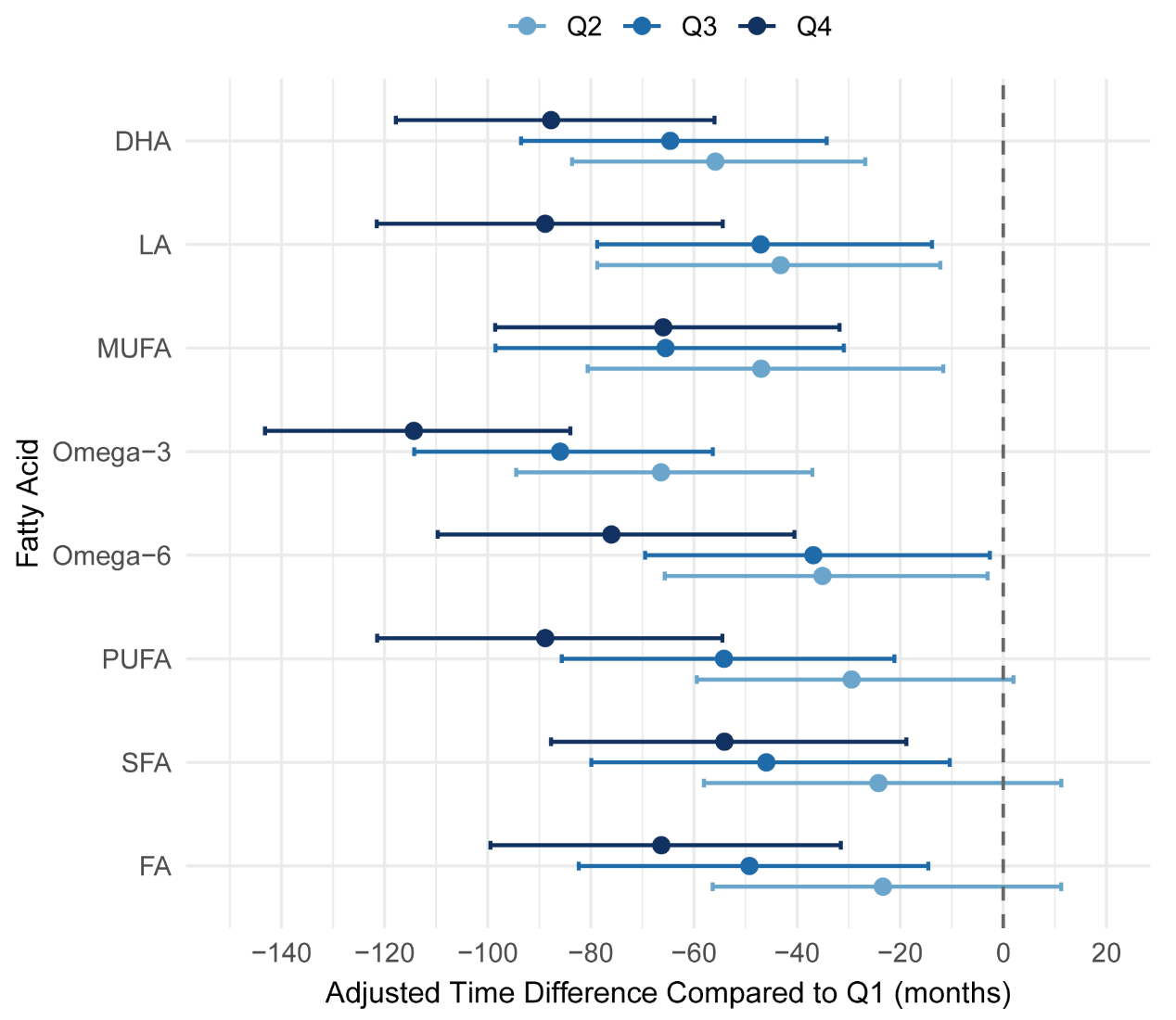


Figure S1. Adjusted median time differences for pulmonary arterial hypertension across fatty acid quartiles. Models were fully adjusted for age, sex, race, Townsend Deprivation Index (TDI), body mass index (BMI), smoking status, diabetes mellitus (DM), hypertension, alcohol consumption, diet score, metabolic equivalent of task (MET), history of cardiovascular disease (CVD), and chronic pulmonary diseases. DHA: docosahexaenoic acid; LA: linoleic acid; MUFA: monounsaturated fatty acids; Omega-3: Omega-3 fatty acids; Omega-6: Omega-6 fatty acids; PUFA: polyunsaturated fatty acids; SFA: saturated fatty acids; FA: total fatty acids.
